# As a downstream target of the AKT pathway, NPTX1 inhibits proliferation and promotes apoptosis in hepatocellular carcinoma

**DOI:** 10.1042/BSR20181662

**Published:** 2019-06-04

**Authors:** Yue Zhao, Yaqi Yu, Wenxiu Zhao, Song You, Min Feng, Chengrong Xie, Xiaoqin Chi, Yi Zhang, Xiaomin Wang

**Affiliations:** 1 Department of Hepatobiliary Surgery, Zhongshan Hospital, Xiamen University, Fujian Provincial Key Laboratory of Chronic Liver Disease and Hepatocellular Carcinoma, Xiamen, Fujian, P.R. China; 2 Faculty of Clinical Medicine, Fujian Medical University, Fuzhou, Fujian, P.R. China

**Keywords:** cancer apoptosis, cancer proliferation, hepatocellular carcinoma, NPTX1, the AKT pathway

## Abstract

Hepatocellular carcinoma (HCC) is correlated with a poor prognosis and high mortality worldwide. Neuronal pentraxin 1 (NPTX1) has been reported to play an oncogenic role in several types of tumors. However, its expression and function in HCC is not yet fully understood. In the present study, we aimed to investigate the clinicopathological significance of NPTX1 in HCC and the underlying mechanisms. We observed that the expression of NPTX1 was decreased significantly in HCC and was associated with tumor size and metastasis in patients. Gain-of-function approaches revealed that NPTX1 suppressed the growth ability of HCC cells and contributed to mitochondria- related apoptosis. Furthermore, mechanistic investigations showed that the AKT (AKT serine/threonine kinase) pathway can regulate the effects of NPTX1 in HCC cells. After blocking the AKT pathway, the action of NPTX1 was greatly increased. In summary, we demonstrated that NPTX1 inhibited growth and promoted apoptosis in HCC via an AKT-mediated signaling mechanism. These findings indicate that NPTX1 is a potential clinical therapeutic target.

## Introduction

Hepatocellular carcinoma (HCC) is the fifth most common cancer and ranked third among causes of cancer-related mortality worldwide [1,2]. Despite the rapid development of HCC treatments, such as surgery, chemotherapy, radiotherapy, and targeted therapy, the prognosis of patients with HCC remains unsatisfactory because of the high rate of intrahepatic recurrence and the lack of effective therapy [3]. Thus, it is urgent to clarify the molecular mechanisms underlying HCC development to identify novel diagnostic markers and promising treatment strategies for improving the prognosis of HCC patients.

As a member of the long pentraxin family of proteins, neuronal pentraxin 1 (NPTX1) is mainly expressed in central neurons and plays roles in promoting neurite outgrowth and modulating cellular properties [4,5]. The long pentraxin family has two other members: neuronal pentraxin 2 (NPTX2) and neuronal pentraxin receptor (NPTXR) [6,7]. Previous studies have reported that NPTX1 acts as a mediator of mitochondria-mediated hypoxic–ischemic neuronal injury via a glycogen synthase kinase 3α/β (GSK3α/β)-dependent mechanism [8–10]. In recent years, an increasing number of studies have shown that NPTX1 may be involved in the progression of cancers, including lung cancer [11], colon cancer [12], and pancreatic cancer [13,14]. For example, the expression of NPTX1 is down-regulated in colon cancer and inhibits cell proliferation via the down-regulation of Cyclin A2 and cyclin-dependent kinase 2 (CDK2) expression. However, the expression pattern and biological function of NPTX1 in HCC remain unclear.

As an oncoprotein in human cancers, the AKT serine/threonine kinase (AKT) plays a key role in cell-to-cell signaling during tumorigenesis [15]. There are numerous downstream targets in the AKT signaling pathway, including GSK-3 and FoxO, which significantly enhance the functionality of AKT. For instance, the activation of AKT causes the phosphorylation of the downstream target GSK-3β [16], thereby decreasing the transcriptional repression of Snail and reducing the degradation of β-catenin [17]. This dynamic and complex network of hyperactivated AKT signaling pathways contributes to cancer cell proliferation, survival and migration. There are three AKT isoforms (AKT1–3), which are encoded by separate genes and share more than 80% identity at the amino acid level. Previous genetic studies in mice demonstrated that AKT1 deletion inhibited cancer development in various mouse models [18–20], whereas AKT2 deletion had limited effects on cancer development in mouse models [21,22]. Interestingly, AKT is highly mutated and activated in various cancers, especially HCC [23]. Previous studies have shown that the AKT cascade participates in HCC progression and is associated with poor prognosis and survival in HCC patients [23,24]. Therefore, further understanding of the mechanisms of the AKT signaling pathway could provide new insight into the etiology of HCC.

Herein, we provide the first report that NPTX1 is down-regulated in HCC and that NPTX1 expression is correlated with tumor size and metastasis. Ectopic expression of NPTX1 suppressed cell proliferation and promoted apoptosis. As a downstream target of AKT signaling, the effects of NPTX1 on HCC are regulated by the AKT pathway. Our investigations revealed the role of NPTX1 in HCC progression.

## Materials and methods

### Human HCC samples

All HCC and corresponding peritumor tissue samples and follow-up information were provided by the Chronic Liver Disease Biological Sample Bank, Department of Hepatobiliary Surgery, Zhongshan Hospital Xiamen University. None of the patients had received any preoperative treatment before undergoing hepatectomy. All the procedures for sample collection were approved by the Ethics Committee of the Zhongshan Hospital of Xiamen University, and written informed consent was obtained from all patients. In this experiment, we used 53 pairs of paraffin-embedded HCC tissue and matched adjacent normal tissue, and we used mRNA from an additional 74 pairs of matched tissues from HCC patients.

### Cell culture

HepG2, BEL-7402, QGY-7701, SMMC-7721, PLC/PRF/5, SK-Hep-1, Huh-7, and LO2 cells were obtained from the Cell Bank of the Chinese Academy of Sciences, and MHCC-97h and HCC-LM3 cells were obtained from Zhongshan Hospital of Fudan University. The cells were cultured in DMEM (HyClone) supplemented with 10% fetal bovine serum (FBS; Gibco) at 37°C in 5% CO_2_.

### Immunohistochemistry

HCC and adjacent normal tissues were fixed with 10% formalin, and embedded in paraffin, and 3-μm-thick sections were made. The sections were deparaffinized, hydrated and soaked in 3% H_2_O_2_ at room temperature for 1 h. After blocking nonspecific binding proteins, the slides were incubated with an NPTX1 polyclonal antibody at 4°C in a moist chamber overnight. The slides were sequentially incubated with a biotinylated secondary antibody and then streptavidin–peroxidase conjugate, each for 30 min at room temperature. Finally, 3,5-diaminobenzidine (DAB) was used for color development, which was followed by Hematoxylin counterstaining.

### Plasmid construction and lentivirus preparation

For NPTX1 overexpression, a 1299-bp genomic sequence of the NPTX1 coding region was inserted into the pLV-puro plasmid (Inovogen Tech, Beijing, China). For NPTX1 knockdown, shRNA targeting NPTX1 was inserted into the pLV-shRNA-puro plasmid (Inovogen Tech, Beijing, China). The target sequence of NPTX1 shRNA is 5′-GATCCGCAAACTTTGCAATCGCTC AACTCGAGTTGAGCGATTGCAAAGTTTGCTTTTTG-3′. HEK 293T cells were transfected with the plasmids mentioned above and the virus packing plasmids by using TurboFect Transfection Reagent (Thermo Scientific). The virus-containing medium was harvested and filtered to remove cell debris after 48 h. HCC cell lines were plated in six-well plates. Twenty-four hours later, the cell lines were transduced with virus-containing medium supplemented with Polybrene (10 µg/ml). Infected cells were cultured for selection with puromycin (InvivoGen) after two infections to establish stably infected cell lines.

### Western blot analysis

Proteins were extracted using RIPA lysis buffer containing a protease inhibitor cocktail (Sigma, St. Louis, MO, U.S.A.) and a phosphatase inhibitor cocktail (Roche, Mannheim, Germany). After quantitation using the Bradford method, protein lysates were subjected to sodium dodecyl sulfate polyacrylamide gel electrophoresis and transferred to a polyvinylidene fluoride membrane (Millipore). The membranes were blocked with nonfat milk for 1 h and incubated with primary antibodies, which are listed in Supplementary Table S1, overnight at 4°C. They were then incubated with goat anti-rabbit (111-035-003, Jackson) or anti-mouse (115-035-003, Jackson) HRP–conjugated secondary antibodies. Protein bands were visualized by enhanced chemiluminescence (Millipore). β-actin was used as a loading control.

### Real-time PCR analysis

Total RNA from the different cell lines, HCC tissues and adjacent normal liver tissues was extracted with TRIzol reagent (Invitrogen) according to the manufacturer’s instructions. cDNA was synthesized using the GoScript™ Reverse Transcription System Kit (Promega, Madison, WI). Quantitative real-time PCR (qRT-PCR) was performed with the Lightcycle 96 Real-Time PCR System (Roche) using FastStart Universal SYBR Green Master (Rox) (Roche). The following primers were used for amplification of NPTX1: sense primer, 5′- GAGACAAGTTCCAGCTCACA-3′, and antisense primer, 5′- CAGACAGTGAAGGCGTACAT-3′. Glyceraldehyde-3-phosphate dehydrogenase (GAPDH) was amplified as an internal control using sense primer, 5′-CGACCACTTTGTCAAGCTCA-3′, and antisense primer, 5′-GGAGAGTCAACGGGCATATAG-3′. Comparative quantitation was determined using the 2^−ΔΔ*C*^_t_ method.

### Cell proliferation and colony formation assay

For the cell proliferation assay, 1 × 10^3^ cells per well were seeded in 96-well culture plates. Cell counting kit-8 (CCK-8) (Dojindo) was added to each well at the indicated time points and incubated at 37°C for 1 h. The absorbance values were measured using a spectrophotometer (Bio-Rad) at a wavelength of 450 nm. For the colony formation assay, 2 × 10^3^ cells per well were seeded in six-well culture plates. After 14 days of culture, the cells were fixed in 4% paraformaldehyde and stained with Crystal Violet.

### Flow-cytometry assay

Control or pLV-NPTX1 SMMC-7721 cells were harvested at the exponential growth phase, and single-cell suspensions were fixed with 70% ethanol. The cell cycle was monitored using propidium iodide (PI) staining and measured with a flow cytometer, and the results were analyzed with ModFit 3.0 software (Verity Software House, Topsham, ME, U.S.A.). Cells were treated with cisplatin (10 µg/ml) 24 h prior to analysis. Apoptosis was detected by using an Apoptosis Detection Kit (Dojindo) according to the manufacturer’s instructions. Stained cells were then analyzed with a flow cytometer, and Kaluza software was used to analyze the data.

### Terminal deoxynucleotidyl transferase-mediated dUTP-biotin nick end labeling assay

For terminal deoxynucleotidyl transferase-mediated dUTP-biotin nick end labeling (TUNEL) analysis, cells were fixed in 4% paraformaldehyde and then analyzed using a DeadEnd colorimetric TUNEL analysis kit (Promega, Madison, WI) according to the manufacturer’s instructions.

### Tumor xenografts in nude mice

A xenograft mouse model was developed using 5- to 6-week-old male BALB/c nude mice. Control or pLV-NPTX1 SMMC-7721 cells were trypsinized and harvested in serum-free DMEM, and 0.15 ml serum-free DMEM containing 1 × 10^6^ cells was injected subcutaneously into the right flank of each nude mouse. Tumor size was measured after 10 days and every 4 days thereafter. Tumor-carrying nude mice were killed 30 days after injecting the cells, and tumors were removed for further analysis. All animal experiments were approved by the Animal Care and Use Committee of Xiamen University.

### Statistical analysis

All experiments were performed in triplicate. Statistical analyses were performed by using SPSS software (version 19.0). Data are shown as the mean ± S.E.M. Differences between groups were analyzed by Student’s *t* test or chi-square test. The Kaplan–Meier method was performed for the analysis of patient overall survival and recurrence-free survival. *P*<0.05 was considered to be statistically significant.

## Results

### Low expression of NPTX1 in HCC and clinical correlation of NPTX1 expression with poor prognosis

**Figure 1:**
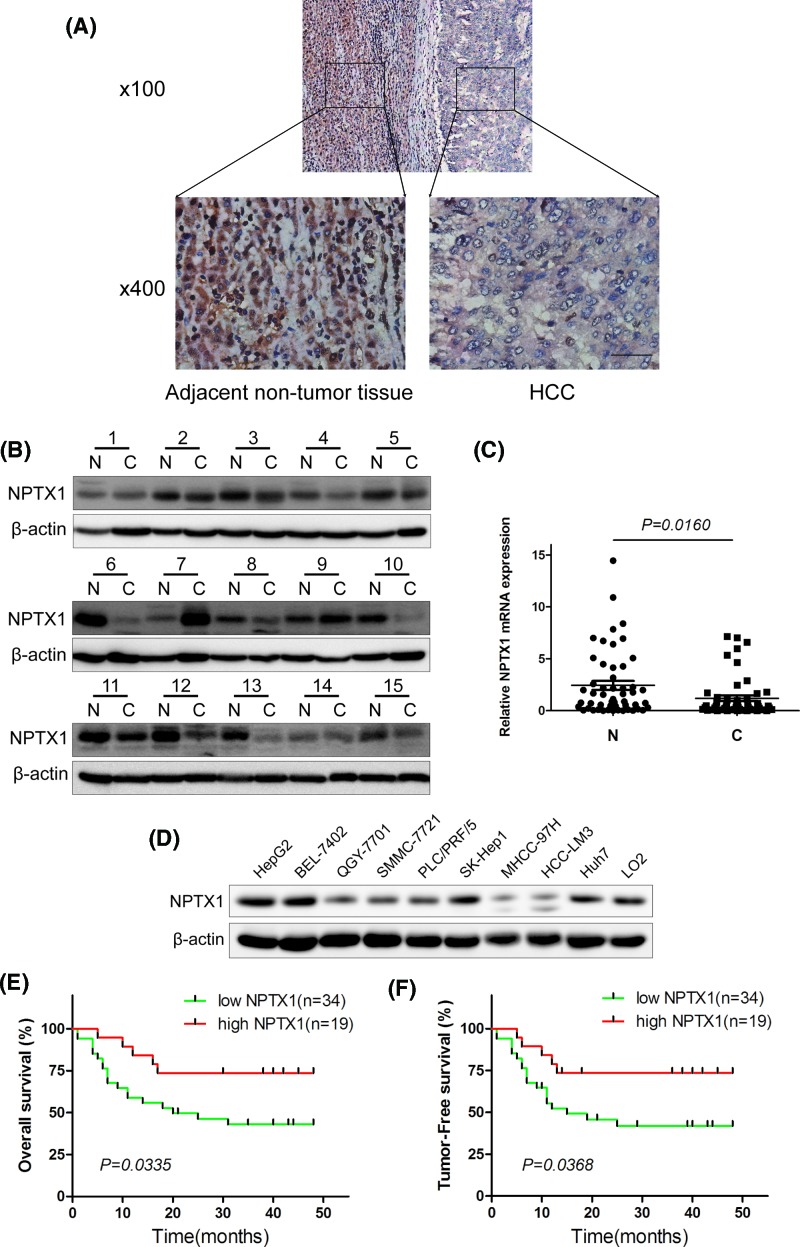
Low expression of NPTX1 is closely correlated with the poor prognosis of patients with HCC (**A**) Immunohistochemistry staining analysis of NPTX1 expression in 53 pairs of cancer tissues and matched adjacent normal tissues. (**B**) Western blot analysis of NPTX1 protein expression in 15 representative HCC tissues (C) and adjacent normal tissues (N). β-actin was used as a loading control. (**C**) NPTX1 mRNA levels were decreased in HCCs relative to the levels in control tissues. (**D**) NPTX1 expression was evaluated in HCC cell lines by Western blot. β-actin was used as a loading control. (**E,F**) Kaplan–Meier analysis of the correlation between NPTX1 expression and overall survival or tumor-free survival of HCC patients.

To investigate the role of NPTX1 in HCC progression, we first examined the expression of NPTX1 in HCC tissues and adjacent nontumor liver tissues by performing immunohistochemical staining. We found that 34 (64.15%) out of 53 HCC specimens showed low expression levels relative to expression in adjacent nontumor tissues, whereas only 19 (35.85%) specimens showed high relative expression levels (Figure 1A). These findings were confirmed by Western blot analysis and real-time RT-PCR (Figure 1B,C). We also detected the expression of NPTX1 in different HCC cell lines and a normal liver cell line (Figure 1D). The cells of the LO2 line are normal liver cells, and they expressed more NPTX1 than did most of the HCC cell lines (QGY-7701, SMMC-7721, PLC/PRF/5, MHCC-97h, HCC-LM3). These results revealed that NPTX1 is down-regulated in HCC.

Then, we analyzed the clinical significance of NPTX1 expression based on clinical data from 53 HCC patients. Statistical analysis revealed that low NPTX1 expression strongly correlated with tumor size (*P*=0.0319) and metastasis (*P*=0.0004), whereas no significant correlations were observed between NPTX1 expression and other factors, such as gender, age, α-fetoprotein (AFP), hepatitis B virus (HBV) DNA copies and differentiation (Table 1). Furthermore, we found by Kaplan–Meier analysis that patients whose tumors showed low NPTX1 expression levels had significantly shorter overall survival and tumor-free survival times than did patients whose tumors showed high NPTX1 expression levels (Figure 1E,F, *P*=0.0335 and 0.0368, respectively). Taken together, these results indicated that down-regulated NPTX1 expression is associated with the clinical progression of human HCC.

**Table 1:** Correlation of NPTX1 protein expression with clinicopathological factors in HCC

Clinicopathological factors	NPTX1 expression		X^2^	*P-v*alue
	High	Low		
Age (years)				
<55	11	15		
≥55	8	19	0.9257	0.3360
Gender				
Male	14	27		
Female	5	7	0.2283	0.6328
AFP (μg/l)				
<400	13	15		
≥400	6	19	2.889	0.0892
HBV DNA copies (cps/ml)				
<1000	7	11		
1000	12	23	0.1095	0.7407
Tumor size (cm)				
<5	10	8		
≥5	9	26	4.603	0.0319*
Differentiation				
Well	1	0		
Moderate	17	27		
Poor	1	7	3.835	0.1470
Metastasis				
Yes	9	31		
No	10	3	12.64	0.0004^†^

*P<0.05. ^†^*P*<0.001.

### NPTX1 inhibits tumor cell proliferation and induces cell cycle arrest

**Figure 2:**
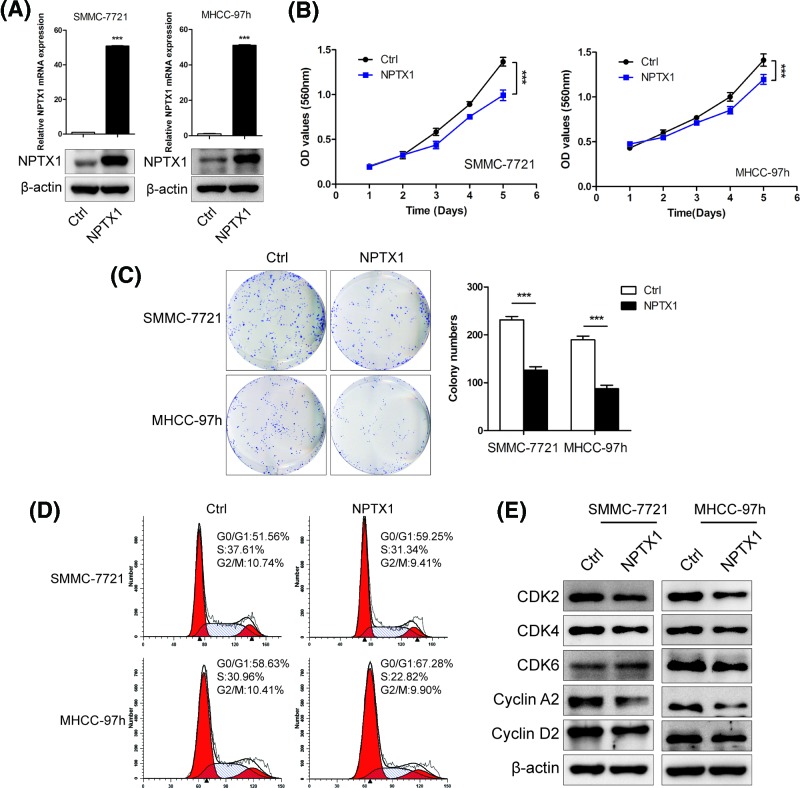
NPTX1 inhibits HCC cell proliferation *in vitro* (**A**) The mRNA and protein levels of NPTX1 in SMMC-7721 and MHCC-97h cells after transfection with lentiviruses expressing NPTX1. (**B**) The proliferation abilities of control and NPTX1-overexpressing cells were assessed by performing a CCK-8 assay. (**C**) Colony formation assays were performed using control and NPTX1-overexpressing SMMC-7721 and MHCC-97h cells. (**D**) The percentage of cells in different phases of the cell cycle was determined by FACS analysis of control and NPTX1-overexpressing cells. (**E**) The protein levels of CDK2, CDK4, CDK6, Cyclin A2 and Cyclin D2 in control and NPTX1-overexpressing cells were measured by Western blot. Data are shown as the mean ± SD; ***P<0.001 (Student’s *t* test). Abbreviation: CDK, cyclin-dependent kinase.

To investigate the biological role of NPTX1 in HCC, we established stable NPTX1-overexpressing SMMC-7721 and MHCC-97h model cells (Figure 2A), which exhibit relatively low expression of NPTX1 among HCC cell lines based on our previous results (Figure 1D). To analyze the effects of NPTX1 on HCC cell proliferation, we performed CCK-8 assays and found that overexpression of NPTX1 suppressed the growth ability of HCC cells relative to that of control cells (Figure 2B). Similarly, NPTX1-overexpressing cells showed a significantly lower colony formation rate than control cells (Figure 2C).

To further reveal the mechanism by which NPTX1 contributes to proliferation, we detected the influence of NPTX1 on cell cycle distribution by performing flow cytometry. NPTX1 overexpression was associated with an increase in the number of HCC cells in the G_0_/G_1_ phase and a decrease in the number of cells entering S phase (Figure 2D), suggesting that NPTX1 could induce G_0_/G_1_ phase arrest in HCC cells. Western blot analysis of CDK2, cyclin-dependent kinase 4 (CDK4), cyclin-dependent kinase 6 (CDK6), Cyclin A2 and Cyclin D2, which are cycle-related proteins, revealed decreases relative to control levels in the expression of CDK2 and Cyclin A2 proteins in NPTX1-overexpressing cells (Figure 2E), whereas there were no significant changes in CDK4, CDK6 and Cyclin D2 levels relative to control levels in either NPTX1-overexpressing cell line. These findings suggest that NPTX1 inhibits cell proliferation by inducing G_0_/G_1_ cell cycle arrest in HCC.

### NPTX1 promotes mitochondria-related apoptosis in HCC cells

**Figure 3:**
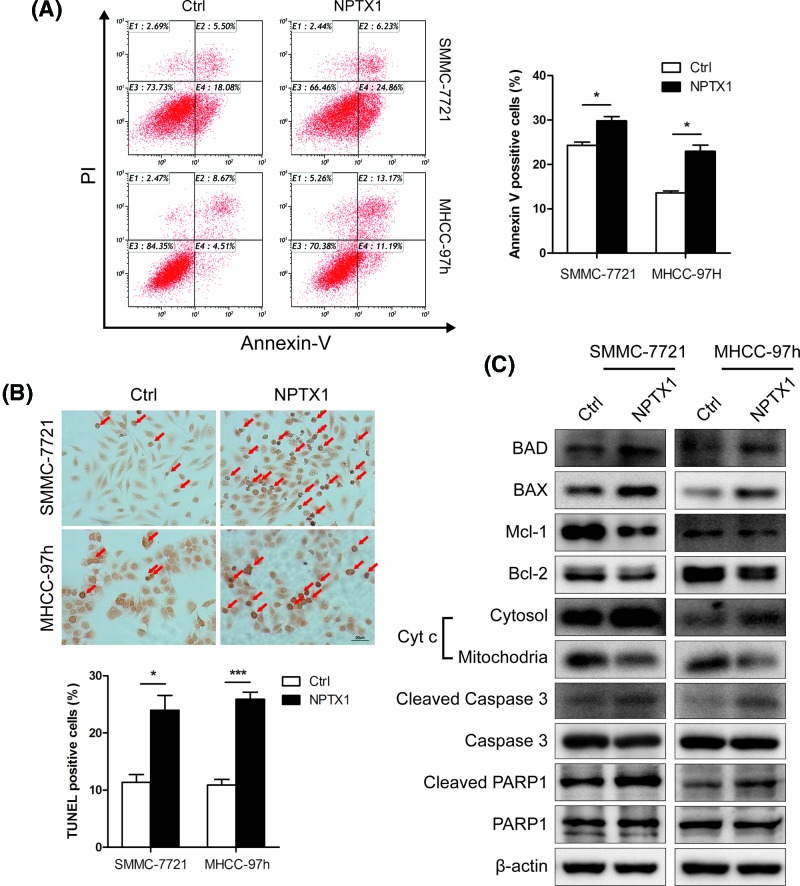
Up-regulated NPTX1 expression induces mitochondria-related apoptosis in HCC cells (**A**) Control and NPTX1-overexpressing SMMC-7721 and MHCC-97h cells were treated with cisplatin (10 µg/ml) for 24 h. After treatment, the cells were analyzed by flow cytometry for Annexin V and PI dual labeling. Annexin V-positive cells were designated as apoptotic cells. The percentage of apoptotic cells is shown. (**B**) Before performing the TUNEL assay, control and NPTX1-overexpressing HCC cells were treated with cisplatin (10 µg/ml) for 24 h. The cells were observed by microscopy at 200× magnification. (**C**) Western blot analysis of BAD, BAX, Mcl-1, Bcl-2, Cyt *c*, Caspase 3 and PARP 1 in control and NPTX1-overexpressing SMMC-7721 and MHCC-97h cells treated with cisplatin (10 µg/ml) for 24 h was performed. Data are shown as the mean ± SD; *P<0.05, ***P<0.001 (Student’s *t* test).

We then analyzed the effects of NPTX1 on apoptosis in HCC cells by performing flow cytometry with Annexin V and PI staining. We observed that NPTX1-overexpressing SMMC-7721 and MHCC-97h cells showed higher proportions of Annexin V-positive cells than did control cells (Figure 3A). This result was further verified by TUNEL assay, which revealed a higher percentage of TUNEL-positive cells among NPTX1-overexpressing HCC cells than among control cells (Figure 3B). Previous reports have shown that, as a mediator of hypoxic injury in the brain, NPTX1 plays a critical role in regulating mitochondria-driven neuron death [8]. We speculated that NPTX1 might contribute to HCC cell apoptosis in a mitochondria-related manner. To test our hypothesis, Western blot analysis of well-known mitochondria-related proteins was performed. We found that the protein levels of BCL2-associated agonist of cell death (BAD) and BCL2-associated X protein (BAX) were increased in NPTX1-overexpressing SMMC-7721 and MHCC-97h cells relative to control cells. In contrast, decreased levels of myeloid cell leukemia sequence 1 (Mcl-1) and B-cell lymphoma-2 (Bcl-2) were found in NPTX1-overexpressing SMMC-7721 and MHCC-97h cells relative to control cells (Figure 3C). Consistently, cytochrome *c* was released from mitochondria to the cytoplasm, and cleavage of caspase 3 and poly ADP-ribose polymerase 1 (PARP1) increased in NPTX1-overexpressing cells, indicating that NPTX1 promotes mitochondria-related apoptosis in HCC cells.

### Ectopic expression of NPTX1 suppresses HCC cell growth and contributes to apoptosis *in vivo*

**Figure 4:**
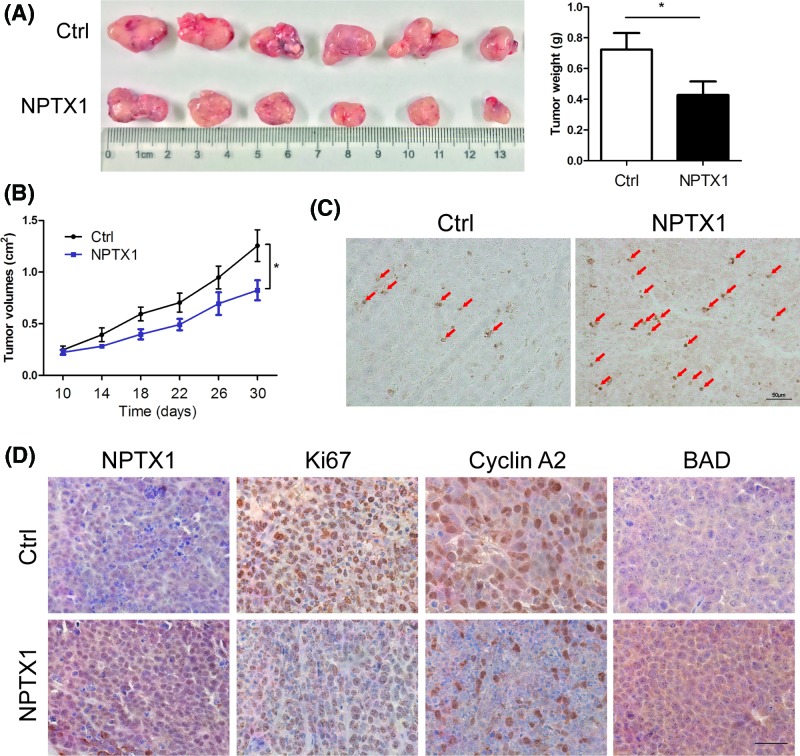
Effects of NPTX1 overexpression on HCC growth and apoptosis *in vivo* (**A**) Control and NPTX1-overexpressing SMMC-7721 cells were injected subcutaneously into the right posterior flank of nude mice. Representative images and tumor weights at 31 days postinjection are displayed. (**B**) Tumor sizes were measured and growth curves were generated (*n*=6). (**C**) TUNEL assays were performed to identify the apoptotic cells in tumor xenografts of nude mice. The results were observed by microscopy at 200× magnification. (**D**) Immunohistochemical staining for NPTX1, Ki67, Cyclin A2 and BAD proteins in tumor xenografts of nude mice. The results were observed by microscopy at 400× magnification. Data are shown as the mean ± SD; *P<0.05 (Student’s *t* test).

To verify the function of NPTX1 in HCC progression, we established xenograft mouse models by subcutaneously injecting control and NPTX1-overexpressing SMMC-7721 cells into nude mice. As shown in Figure 4A, compared with control cells, NPTX1-overexpressing SMMC-7721 cells generated smaller volumes and weights of xenografts in nude mice. We also observed that xenografted tumors derived from NPTX1-overexpressing SMMC-7721 cells grew slower than did tumors derived from control cells (Figure 4B). To examine the effects of NPTX1 on apoptosis in xenografted tumors, a TUNEL assay was performed to detect the apoptosis rates in control and NPTX1-overexpressing xenografts. We found more TUNEL-positive cells in NPTX1-overexpressing xenografts than in control xenografts (Figure 4C). Subsequently, immunohistochemistry staining analysis revealed weaker staining for Ki67 and Cyclin A2 in NPTX1-overexpressing xenografts than in control xenografts (Figure 4D). In contrast, stronger BAD expression was observed in NPTX1-overexpressing xenografts than in control xenografts, consistent with our previous results. Collectively, our findings suggest that NPTX1 inhibits tumor growth and promotes apoptosis *in vivo*.

### AKT acts as an upstream factor of NPTX1 and inhibits the effects of NPTX1 in HCC cells

**Figure 5:**
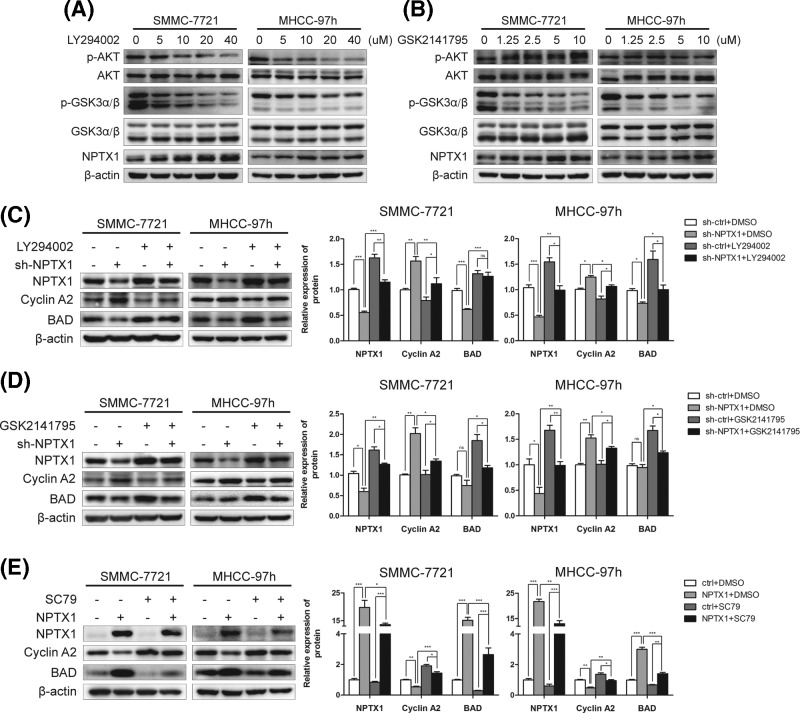
The AKT pathway is involved in the NPTX1-mediated functions in HCC cells (**A,B**) The protein levels of p-AKT, total AKT, p-GSK3α/β, total GSK3α/β, and NPTX1 in SMMC-7721 and MHCC-97h cells were measured by Western blot analysis after incubation with LY294002 for 1 h and with GSK2141795 for 4 h at the indicated doses. (**C,D**) Western blot analysis of NPTX1, Cyclin A2 and BAD in sh-NPTX1 SMMC-7721 and MHCC-97h cells after treatment with LY294002 (20 µM) for 1 h and GSK2141795 (5 µM) for 4 h was performed. (**E**) Western blot analysis of control and NPTX1-overexpressing HCC cells after incubation with SC79 (10 µg/ml) for 2 h was performed. β-actin was used as a loading control. ImageJ software was used to quantitate the protein bands. Data are shown as the mean ± SD; *P<0.05, **P<0.01, ***P<0.001 (Student’s *t* test).

As an oncogene reported to play a critical role in HCC progression, AKT regulates various cellular functions, including proliferation, apoptosis and invasion [25,26]. To investigate the potential molecular mechanisms linking NPTX1 and the AKT pathway, we treated SMMC-7721 and MHCC-97h cells with the phosphoinositide-3-kinase inhibitor LY294002. We found that the levels of phosphorylated AKT and phosphorylated GSK3α/β were significantly reduced after treatment with LY294002, whereas the expression of NPTX1 was enhanced by LY294002 treatment in a dose-dependent manner (Figure 5A). We also observed a similar dose-dependent NPTX1 regulation trend in HCC cells treated with the AKT inhibitor GSK2141795 (Figure 5B), which indicate that the expression of NPTX1 might be modulated by the AKT signaling pathway in HCC.

To determine whether AKT functions upstream of NPTX1 to regulate cell cycle-related proteins and mitochondria-related proteins in HCC cells, we treated NPTX1 knockdown HCC cells with the inhibitors LY294002 and GSK2141795. LY294002 and GSK2141795 rescued the decreased NPTX1 expression in NPTX1 knockdown cells, thereby inhibiting the up-regulated expression of Cyclin A2 induced by NPTX1 knockdown and rescuing the suppression of BAD induced by NPTX1 knockdown (Figure 5C,D). In contrast, SC79, an AKT phosphorylation activator, inhibited the up-regulated NPTX1 expression in NPTX1-overexpressing cells, thereby reversing Cyclin A2 and BAD expression levels in NPTX1-overexpressing cells (Figure 5E). These results indicate that AKT signaling pathway modulated downstream targets, such as Cyclin A2 and BAD, via regulating the expression of NPTX1 in HCC cells.

## Discussion

The result of the present study showed that the expression of NPTX1 was decreased in HCC specimens. A similar result was reported in colon cancer [12]. However, the present study is the first to show that NPTX1 expression level correlates with clinicopathological factors (tumor size and metastasis) in HCC and is associated with survival time in HCC patients. Previous studies have identified NPTX1 as an epigenetic target and showed that it acted as a methylation marker in human pancreatic cancer [27], and consistent results were also have been found in cervical cancer [28] and colorectal cancer [29]. In 2015, Zhou et al. [11] demonstrated that promoter hypermethylation contributes to lower NPTX1 expression in lung cancer, which may lead to cancer pathogenesis. In addition, NPTX1 was found to be an epigenetic target in regulating HDAC3-mediated neurotoxicity [30]. All these findings indicated that epigenetic regulation may be an important reason why NPTX1 shows low expression in various cancers, and ultimately contributes to the poor prognosis.

The high proliferation ability and resistance to death of cancer cells are worldwide obstacles in cancer therapy, and these features contribute to cancer recurrence [31]. In our study, we found that NPTX1 inhibits cell proliferation by inducing cell cycle arrest and down-regulating cycle-related proteins (Cyclin A2 and CDK2), these findings are consistent with findings in colon cancer [12]. Cyclins play a critical role in modulating the cell cycle through binding and activating cyclin-dependent kinase (CDK) enzymes [32,33]. As the regulatory subunit of CDK2, Cyclin A2 is essential for cycle phase transitions and could be an independent prognostic factor for the relapse of human HCC [34,35]. NPTX1 inhibits the level of Cyclin A2 in HCC, implicating an anti-cancer role of NPTX1 in cancer progression. Unexpectedly, we did not observe any changes in the levels of CDK4, CDK6 and Cyclin D2 proteins, which indicated that NPTX1 did not modulate cell proliferation via the CDK4 or CDK6/Cyclin D complex pathway.

Previous work showed that after exposure to hypoxia, NPTX1 was induced in a time-dependent manner and that this induction preceded neuronal death in the neonatal brain, suggesting that NPTX1 acts as an apoptosis mediator during brain injury [9,10]. Interestingly, subsequent studies reported that NPTX1 was involved in regulating apoptosis in various cells, such as pancreatic β-cells [36] and human endometrial endothelial cells [37]. However, the effects of NPTX1 on apoptosis in HCC remain unclear. Our present study revealed that NPTX1 could contribute to apoptosis in HCC. Moreover, we found that NPTX1 promotes apoptosis in the mitochondria-related apoptotic pathway. Mitochondrial impairment or dysfunction could rapidly induce the inhibition of cell survival, and mitochondrial activity-related therapies often achieve satisfying results [38,39]. Thus, NPTX1 represents a potential therapeutic target in clinical treatment.

AKT plays indispensable roles in cellular proliferation, apoptosis, and invasion in various kinds of tumors, promoting cancer progression as an oncogene [25,26]. In HCC, AKT signaling has been identified as an important mediator of multiple functions [24]. Our present study demonstrated that AKT signaling can down-regulate the expression of NPTX1 and modulate its function, thereby being responsible for proliferation and apoptosis in HCC. The balance between growth and apoptosis is usually controlled by the AKT pathway. AKT promotes cell survival by inhibiting apoptosis through its ability to activate or inactivate several targets, including BAD and caspase 3 [40]. The present results showed that AKT signaling could abolish the effects of NPTX1 on Cyclin A2 and BAD, confirming the regulatory role of AKT in HCC. AKT is significantly activated in HCC specimens [23], which explains why NPTX1 shows low expression in HCC. The AKT signaling pathway mediates the expression and stabilization of methyltransferase in various cancers and promotes tumor progression [41,42]. Based on previous studies that identified NPTX1 as an epigenetic target, we speculate that in HCC, the AKT signaling pathway down-regulates NPTX1 by modulating the potential methyltransferases that catalyze the methylation of NPTX1. This hypothesis requires further investigation. Although down-regulated expression of NPTX1 occurs in diverse tumor types, the function and molecular mechanisms are not fully understood. Whether NPTX1 performs its function in other malignancies through the AKT signaling pathway warrants exploration.

## Conclusion

In summary, we revealed that NPTX1 is down-regulated in HCC and that its expression is associated with clinicopathological factors. Furthermore, we identified NPTX1 as an important regulator of HCC cell proliferation and apoptosis via AKT signaling. These findings indicate that NPTX1 can be utilized as a prognostic marker and potential therapeutic target.

## Supporting information

**Supplementary Table S1:** Information on antibodies used for the correlation analysis

## Abbreviations

AKT: AKT serine/threonine kinase
BAD: BCL2-associated agonist of cell death
CCK-8: cell counting kit-8
CDK2: cyclin-dependent kinase 2
CDK4: cyclin-dependent kinase 4
CDK6: cyclin-dependent kinase 6
FoxO: forkhead transcription factors of the O class
GSK3α/β: glycogen synthase kinase 3α/β
HCC: hepatocellular carcinoma
HDAC3: histone deacetylase 3
HRP: horseradish peroxidase
NPTX1: neuronal pentraxin 1
NPTX2: neuronal pentraxin 2
PARP1: poly ADP-ribose polymerase 1
PI: propidium iodide
RIPA: radio immunoprecipitation assay
TUNEL: terminal deoxynucleotidyl transferase-mediated dUTP-biotin nick end labeling
