# Supplementary material for: As a downstream target of the AKT pathway, NPTX1 inhibits proliferation and promotes apoptosis in hepatocellular carcinoma
Source: Biosci Rep. 2019 Jun 4;39(6):BSR20181662. doi: 10.1042/BSR20181662 (PMC6549097; doi:10.1042/BSR20181662)
Supplement: Supplementary file 1 [file bsr20181662_Supp1.pdf]

Supplementary Table

Information on antibodies used for the correlation analysis.

Antibody	WB	IHC	Specificity	Company
NPTX1 (PA5-25954)	1:1000	1:500	Rabbit polyclonal	Thermo Fisher
β-actin (0446WS)	1:2000	/	Mouse monoclonal	CMCTAG
CDK2 (10122-1-AP)	1:1000	/	Rabbit polyclonal	Proteintech
CDK4 (11026-1-AP)	1:1000	/	Rabbit polyclonal	Proteintech
CDK6 (14052-1-AP)	1:1000	/	Rabbit polyclonal	Proteintech
Cyclin A2 (18202-1-AP)	1:2000	1:1200	Rabbit polyclonal	Proteintech
Cyclin D2 (#3741)	1:1000	/	Rabbit polyclonal	Cell Signaling Technology
BAD (10435-1-AP)	1:1000	1:500	Rabbit polyclonal	Proteintech
BAX (50599-2-Ig)	1:1000	/	Rabbit polyclonal	Proteintech
Bcl-2 (#2870)	1:1000	/	Rabbit polyclonal	Cell Signaling Technology
Mcl-1(16225-1-AP)	1:1000	/	Rabbit polyclonal	Proteintech
Cyt C(10993-1-AP)	1:1000	/	Rabbit polyclonal	Proteintech
PARP 1 (13371-1-AP)	1:500	/	Rabbit polyclonal	Proteintech
Caspase-3 (#9662)	1:500	/	Rabbit polyclonal	Cell Signaling Technology
p-AKT (#4060)	1:1000	/	Rabbit monoclonal	Cell Signaling Technology
AKT (#4691)	1:1000	/	Rabbit monoclonal	Cell Signaling Technology
p-GSK3β (#8566)	1:1000	/	Rabbit monoclonal	Cell Signaling Technology
GSK3β (#5676)	1:1000	/	Rabbit monoclonal	Cell Signaling Technology
Ki67(27309-1-AP)	/	1:500	Rabbit polyclonal	Proteintech
